# Consumer or Patient Determinants of Hospital Brand Equity—A Systematic Literature Review

**DOI:** 10.3390/ijerph19159026

**Published:** 2022-07-25

**Authors:** Hanna Górska-Warsewicz

**Affiliations:** Department of Food Market and Consumer Research, Institute of Human Nutrition Sciences, Warsaw University of Life Sciences (WULS), Str. Nowoursynowska 166, 02-787 Warsaw, Poland; hanna_gorska_warsewicz@sggw.edu.pl

**Keywords:** hospital brand equity, health, hospital, perceived quality, systematic literature review, PRISMA

## Abstract

The purpose of this study was to analyze consumer or patient determinants of hospital brand equity (HBE) based on the Preferred Reporting Items for Systematic Reviews and Meta-Analysis (PRISMA) Statement. A search of six databases: Scopus, Web of Sciences, PubMed, Google Scholar, Ebsco, and Elsevier was conducted. A search for studies published up to January 2022 was performed between 15 February and 5 March 2022. Article type, peer-reviewed papers, and studies based on empirical research were used as inclusion criteria. Non-English language papers, dissertations, short reports, works in progress, conference publications, and book chapters were excluded. As a result, a final set of 32 studies were selected for the analysis. Three research questions were formulated on the main determinants of HBE, brand-related factors, and specific medical-related factors. The studies included in the systematic literature review were analyzed in three areas: study description, key findings, and practical recommendations. Among the traditional HBE factors, brand loyalty has been analyzed most often, and the following have also been studied: perceived quality, brand associations, brand awareness, and brand image. Patient satisfaction, service quality, perception of the treatment process, and the work of medical staff were found to be specific medical-related factors. Other factors related to the management process, brand, and patients were also identified. It was noted that the number and variety of medical and other determinants of HBE have increased in recent years. The results of this systematic literature review are relevant to the analysis of consumer/patient behavior in choosing a hospital or other health care facility as they provide a deeper understanding of the increasingly differentiated needs of patients and the way in which the quality of health care services is evaluated.

## 1. Introduction

Hospital brand equity (HBE) is an emerging issue of growing importance [1]. This has been observed in recent years [2,3,4,5,6], although the concept of brand equity has been developed in the literature since the 1980s [7]. This is due to several reasons.

Firstly, previously objective criteria including mortality and morbidity rates were used to evaluate hospital performance. However, with changing customer expectations, subjective customer-centric assessments involving quality [8,9], satisfaction [9,10,11,12,13], and choice [9,14] are also being used to evaluate performance. In addition, as consumer awareness of their rights increases, the patient as a healthcare consumer expects and demands high-quality healthcare [15]. To achieve high healthcare performance, some hospitals have entrenched themselves in the mental maps of patients and communities. However, many hospitals have not achieved this. One reason is that their attempt to improve the quality of healthcare service has been based primarily on investment in advanced medical equipment rather than on a mechanism for continuous quality improvement incorporated into clinical management [16].

Secondly, the role of hospitals has been expanded, crossing the boundaries of medical treatment into health care and public health [17]. In recent years, the development of hospital services is growing and developing rapidly, and this is characterized by the emergence of several types of hospitals, clinics, and other health services, from basic and simple health services to complete and modern health services [18]. Services can also range from general wellness, health screenings, specialist care, and emergency care to chronic illnesses care. Additionally, it is important for hospitals offering these services to ensure the quality of services provided to enable the facility to sustain operations, especially in growing brand equity [19].

Thirdly, dynamic changes in the local and global environment have led to the paradigm shift in the management of public and private hospitals. Hospital management requires an understanding of the needs and desires of patients as a strategy for retaining hospital customers. Competition between hospitals in attracting patients is no longer limited to the functional attributes of the services provided, but rather is related to the perception of the health service [20]. The hospital, as a service provider, must have a good understanding of consumer expectations and desires to provide the services expected by patients [18]. This is what an understanding of HBE can provide.

Fourthly, BE reflects perceived value as seen through the eyes of the patient, so hospitals need to establish a platform for consumer/patient relationships [21,22,23]. This will generate positive emotions towards the hospital [24] and ensure a place in the consumer’s heart (positioning) [18]. It will also strengthen patient trust [18,25] and increase the prestige of the hospital in the eyes of consumers [18] due to the high interaction between the customer and the healthcare provider, i.e., between the patient and the hospital [26].

The above arguments are valid for both public and private hospitals, but for public hospitals, additional issues should be noted. Brand equity is essential in government sectors because it can increase the public’s credibility, trust, and loyalty to the government [27]. Moreover, despite the importance of the public health sector, there has been criticism that the industry suffers from lower customer satisfaction due to a lack of understanding of customers’ needs from a marketing perspective [28]. Gaining a deeper understanding of HBE may help illuminate areas where such institutions as healthcare will improve service quality [16], better recognize patient needs [18], and not waste resources carrying social and economic costs [4]. This will enable hospitals to implement their strategies, convey an increasing range of services, and thus generate profits [21].

For this reason, this study aimed to analyze the consumer or patient determinants influencing HBE based on the PRISMA systematic literature review (SLR).

The following research questions were posed:

- What factor in determining HBE is the most commonly studied in relation to hospitals?
- Are traditional determinants (brand loyalty, perceived quality, brand image, brand associations, brand awareness, and brand familiarity) analyzed in empirical research on HBE?
- What specific medical-related determinants contribute to HBE?

This research fills a gap in the current state of knowledge on BE in healthcare by identifying factors determining HBE, as well as pointing to practical recommendations. Taking only empirically based studies for analysis, this SLR fills the gap of theoretical/practical relevance. Today, this is important in many areas, as practical relevance is important both at the stage of knowledge production and transfer [29]. Therefore, the first research question is to indicate what factor is most often analyzed as a determinant of HBE. In the second question, it is important to identify which traditional HBE determinants proposed in theoretical models are studied in the case of hospitals. The goal of the third research question is to identify hospital-specific factors that, seen through the eyes of patients, determine HBE.

The structure of the article is as follows. Section 2 discusses a literature review on brand equity and its components. Section 3 presents the research questions and discusses the methodology of the systematic literature review. Section 4 contains a discussion of the results related to the general characteristics of the studies included in the SLR, the identification of factors, their description, and practical recommendations. The last two sections present a discussion of the results including suggested directions for future research and conclusions.

## 2. Literature Review

### 2.1. Defining Brand Equity in Hospital Contexts

The first definition of BE was published by P.H. Farquhar and indicates the added value that a brand gives to a product. Three elements of BE were defined: positive brand evaluation, accessible brand attitudes, and a consistent brand image that is relevant from the perspective of the consumer, company, and trade. Thus, by a brand that is well perceived and well evaluated by consumers, an organization, institution, or company can obtain higher prices, reduce marketing costs, and exploit competitive advantages, which affects cash flow [7]. This approach has become the basis for the two most frequent concepts by D.A. Aaker [30] and K.L. Keller [31]. D.A. Aaker defined BE as the set of brand assets and liabilities associated with an organization’s name and symbol that add or subtract value delivered by a product or service [30]. In this view, BE consists of five elements, including brand loyalty, brand awareness, perceived quality, brand associations, and other proprietary brand assets [30]. K.L. Keller analyzed BE from a consumer perspective as consumed-based brand equity indicating the differential impact of brand knowledge on consumer response to the marketing of that brand. In this view, brand knowledge encompasses brand awareness and brand image. Brand image is created by brand associations, particularly their uniqueness, type, strength, and favorability [31]. He proposed a model consisting of four complex elements: (1) brand identity including brand salience and brand awareness, (2) brand meaning, with brand performance and brand imagery, related to brand associations, (3) brand responses with consumer feeling and judgments, and (4) brand resonance, including brand loyalty [31].

Regarding hospitals, brand equity is defined in a manner analogous to that of D.A. Aaker [30] or K.L. Keller [31], indicating a consumer or patient perspective. What differs is the identification of the factors that determine it and the definition of its importance. However, as indicated earlier, brand equity can be important in health service contexts, especially in hospitals, where it can be used to improve service quality [16], better recognize patient needs [18], and ensure patient satisfaction [9,10,11,12,13]. For the consumer, BE provides intangible value by enhancing the interpretation and processing of service information [30]. For an institution, having high brand equity means more cost-effective activities and achieving a competitive advantage [30], which positively affects financial performance [32,33].

### 2.2. Traditional Elements Determining Brand Equity

The two theoretical concepts of BE described above were analyzed for different categories of products and services [34,35,36,37,38,39]. For example, among products, BE has been studied for TV sets [38,40] and cars [40], and among services, for hotels [41,42,43,44,45,46,47,48,49], airlines [50,51,52], and restaurants [48,53,54,55]. Franchise-based BE [56], attendee-based brand equity [57,58], destination BE [59,60,61], place BE [62,63], and city BE [64,65,66] were also studied. In these studies, BE was adapted considering the specifics of the product and service categories studied.

Based on the above analysis and previous research [65,67,68], it was assumed that the traditional elements that make up BE are brand loyalty, perceived quality, brand associations, and brand awareness from D.A.’s concept [30] and brand knowledge, brand awareness, and brand image from K.L. Keller’s concept [31]. A repeated element in both concepts is brand awareness, but it should be noted that brand associations are not mentioned directly in K.L. Keller’s concept, but in the detailed explanations, it is indicated that brand associations determine the brand image. The approach to loyalty is also interesting. In Keller’s concept, brand loyalty is the outcome of BE [31], whereas in Aaker’s model, brand loyalty is one of the equivalent elements that comprise and shape BE [30]. The traditional elements of BE are discussed below.

Brand loyalty can be defined as a positively directed response to a brand [69] or a psychological commitment to purchase again, despite situational influences and marketing activities that could potentially cause a behavior change [70]. Brand loyalty can also be analyzed as a behavioral approach [71], in terms of attitude [72,73] and multidimensionally [69,73,74]. In the behavioral approach, brand loyalty refers to consumers’ repeated choices [71], whereas attitude loyalty is related to consumers’ preferences, commitment, or purchase intentions [72,73]. The multidimensional approach to brand loyalty refers to repeated buying behavior for a set of alternative brands using psychological processes (decision making, evaluation) [74,75].

Brand quality, as with perceived quality, is defined as a consumer’s assessment of the overall superiority or excellence of a product/service. This should be understood as consumers’ subjective assessment of a product, rather than objective quality, based on their perceptions [76]. The perceived quality is also the customer’s perception of the overall quality, superiority, or excellence of the product or service concerning their intended purpose, compared to alternatives [30].

Brand knowledge includes brand awareness and brand image. Brand awareness is considered as familiarity, content/engagement signal, or an “anchor to which other associations can be attached” [30]. It is also analyzed as an essential element of image creation [31], representing the brand in the mind of the target audience [77]. In turn, brand associations are defined as the brand’s assets and liabilities which are linked to the memory of the consumer [30] or as “informational nodes linked to the brand node in memory and [they] contain the meaning of the brand for consumers” [78]. Brand associations are linked to three characteristics named attributes, benefits, and attitudes [31]. They can be analyzed as a separate element of BE or as an element that determines the brand image, and thus constitutes BE. From this point of view, brand image is described as a distinct set of associations related to a brand that is in the memory of the consumer or customer [59]. Similar definitions apply to the set of perceptions about a brand reflected by associations about the brand in a consumers’ memory [31] and the set of beliefs, perceptions, and impressions that a person has about an object [79]. It is worth quoting two other definitions of brand image relating to the overall perception of the brand, based on the information about the brand and experience [80] and the public’s overall impression of a company or its brand [81].

## 3. Materials and Methods

### 3.1. Study Design

The SLR was conducted using the Preferred Reporting Items for Systematic Literature Reviews and the Meta-Analyses (PRISMA) method [82,83]. This research method was chosen because of its importance in many academic studies [65,67,68,84,85,86].

### 3.2. Conducting the SLR

SLR was conducted in six databases: Scopus, Web of Science, PubMed, Google Scholar, Elsevier, and EBSCO. The databases were searched between 15 February and 5 March 2022 for studies published up to January 2022. Inclusion and exclusion criteria were adopted. Article type, peer-reviewed papers, and studies based on empirical research were used as inclusion criteria. No time restrictions were applied.

Non-English language, doctoral dissertations, short reports, works in progress, conference publications: proceedings, posters, abstracts, and book chapters were used as exclusion criteria.

### 3.3. Search Strategy

The following search strategies were applied:

- Scopus: TITLE—ABS—KEY (hospital AND brand AND equity), TITLE—ABS—KEY (hospital AND (brand AND equity)) TITLE—ABS—KEY (healthcare AND brand AND equity)
- Web of Sciences: ALL FIELDS: (hospital) *AND* ALL FIELDS: (brand) *AND* ALL FIELDS: (equity); ALL FIELDS: (healthcare) *AND* ALL FIELDS: (brand) *AND* ALL FIELDS: (equity)
- PubMed: All fields: “hospital brand equity”; All fields: “healthcare brand equity”
- Google Scholar: all in title: hospital brand equity; with the statement: “hospital brand equity”; all in title: healthcare brand equity; with the statement: “healthcare brand equity”
- Elsevier: Title, abstract, keywords: “hospital brand equity”; Title, abstract, keywords: “healthcare brand equity”
- EBSCO: hospital AND brand AND equity; healthcare AND brand AND equity

**Figure 1:**
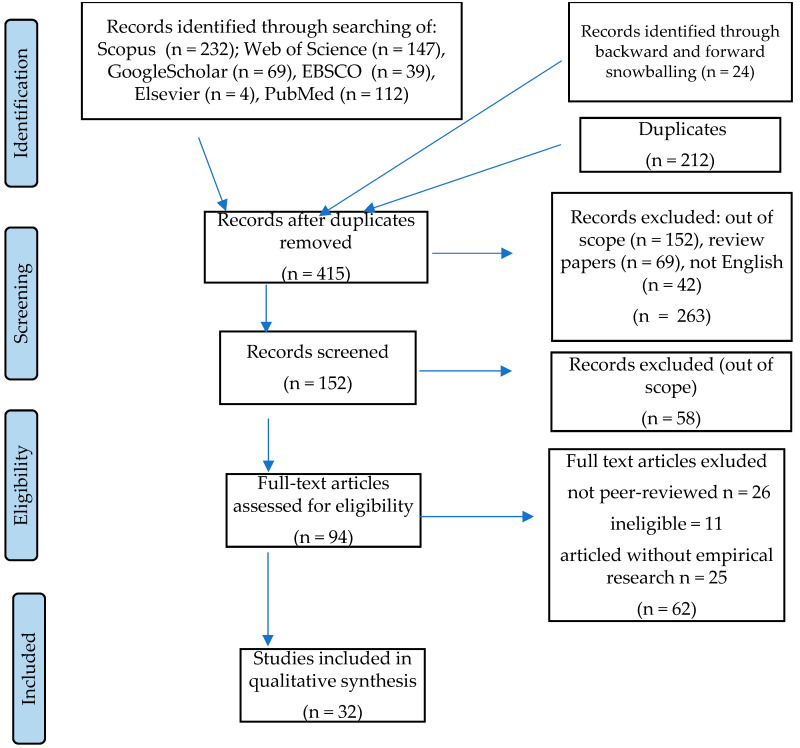
The procedure of identification, screening, eligibility assessment, and inclusion within the systematic review (PRISMA). Source: [82,83].

A search of six databases resulted in the selection of 627 studies. The backward and forward snowball method was also applied, and 24 records were obtained. The backward snowball method is based on checking the references in the papers analyzed. The forward snowball method involves identifying new studies that cite the papers analyzed in the systematic review [87]. After deleting duplicates, 415 records were obtained. The records were then screened based on inclusion and exclusion criteria by title and abstract. The scheme related to identification, screening, assessment of eligibility, and inclusion is shown in Figure 1.

### 3.4. Data Presentation

The analysis of the results was conducted in three sub-sections: details of studies, main findings, and practical recommendations. The following elements are presented in the first part of the description of results: author/s, year of publication, country of study, sample population, and statistical methods. The year of publication and the country in which the study was conducted are presented in graphic form.

The second subsection summarizes the traditional, medical-related, and other determinants of HBE. In the case of HBE determining factors/variables, traditional variables/factors, medical-related variables/factors, and others were distinguished. As traditional variables/factors of HBE, brand loyalty, perceived quality, brand image, brand awareness, brand association, and brand familiarity were identified. They are the result of two main concepts of D.A. Aaker and K.L. Keller, which were described in detail in the second section. As medical variables determining HBE, all variables relating to the treatment process and procedures, hospital equipment, functioning of hospitals as a medical service provider, and all issues relating to medical staff in the context of the provision of medical services and patient relations were accepted.

To summarize, a map of factors was made by listing factors directly and indirectly influencing HBE having a significant statistical impact and those for which no impact was found. Since a solution was applied due to different research methods, different statistical tools, and different research scales, we could not use methods typical of meta-analysis [87,88]. A comparison was made within possible ranges, i.e., within the same statistical tools. The third subsection summarizes practical recommendations by groups of HBE determinants.

## 4. Results

The description of 32 studies that included the SLR is presented in this Section and divided into three sub-sections: Section 4.1—details of studies, Section 4.2—key findings, and Section 4.3—practical implementation.

### 4.1. Details of Studies Included in the SLR

**Figure 2:**
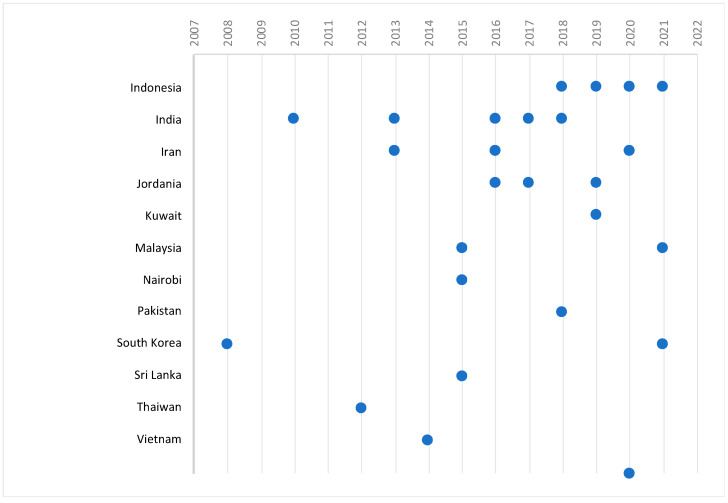
Studies included in the SLR by countries and years of publication.

In this SLR, 32 studies published between 2008 and 2021 were included. Most studies were published in 2018—five studies [9,18,20,89,90] and 2016—four studies [91,92,93,94] (Table 1, Figure 2). Three publications each were published in 2021, 2020, 2019, 2017, and 2015. The studies were conducted in different countries: Malaysia [6,19], South Korea [1,95], Iran [4,91,92,94,96], Indonesia [2,5,18,20,97], Kuwait [98], Jordan [93,99,100,101], India [9,17,23,26,89,102], Pakistan [90], Sri Lanka [103], Nairobi [104], and Vietnam [16,105]. Concentration of research is observed in developing countries, which can be explained by the desire for rapid development on the one hand, and the willingness to compare with highly developed countries on the other. The number of respondents varied significantly and ranged up to 100 people—in four studies [2,20,89,104], 101–250 people—in seven studies [5,19,23,92,97,98,103], 251–500 people—in sixteen studies [3,4,5,6,16,17,90,91,93,94,96,99,100,101,102,106], and over 501 people—in five studies [1,9,18,26,105]. Various methods of statistical analysis were used, including regression analysis [2,18,19,103,104], structural equation modeling (SEM)—in twenty-one studies [1,3,4,6,9,16,17,23,26,90,91,93,94,95,96,97,99,100,101,105,106], multiple logistic regression—in two studies [5,99], descriptive analysis—in two studies [20,98], factor analysis—in two studies [92,102], and cluster analysis—in one study [89].

### 4.2. Main Findings

Table 2 presents the main findings related to the determinants of HBE, divided into traditional, medical-related, and other factors.

As traditional determinants of HBE, brand loyalty [1,4,5,6,18,19,23,26,89,90,91,93,96,97,102,106], perceived quality [6,19,23,26,90,91,97], brand awareness [18,89,90,96,97,106], brand association [18,26,89,91,97,106], and brand image [19,23,90,95] were analyzed. In addition, studies have shown that hospital brand image positively influences patient satisfaction and service quality [2]. In one study, brand image, brand awareness, and brand associations showed no relationship with BE [6].

In the group of medical determinants of HBE, those factors were analyzed that directly related to the treatment process, medical services, and their quality, as well as the patient–hospital staff relationship. Medical services were analyzed through service quality, either patient care service quality with tangibles, reliability, responsiveness, assurance, and empathy [100], as well as the 5Qs model of health-care service quality (HCSQ) with four dimensions as the quality of an object, treatment process, infrastructure, interaction, and the atmosphere [90]. This is an evaluation of medical services by patients and indicates patients’ perceptions of the quality of services provided by the hospital. In this context, service quality was perceived as a critical source of overall BE [99,100,103,106].

Medical staff and the process of providing medical services were also included [3]. The effect on HBE of such factors as first-aid activities, disaster response activities, educational activities, and medical treatment in emergency rooms was studied [95]. The physical environment described by the atmosphere, tangibles, infrastructure facility as well as interpersonal care activity (interaction activity, relationship activity, physician’s care [9] was also analyzed. Customer experience was considered in four dimensions as sensory, affective, behavioral, and intellectual experience [9]. Patient satisfaction was analyzed from the point of view of meeting patients’ needs in the context of their expectations. Among other things, the satisfaction with nurse service, the satisfaction with the use of medical instruments, the satisfaction with administrative service, and the desire to reuse the hospital’s service were considered [2].

One more HBE determinant was clinical governance described as a system through which NHS (National Health Service) organizations are accountable for continually improving the quality of their services and safeguarding high standards of care [16].

Other determinants of HBE include both brand-based factors (other than the traditional ones), as well all activities related to the administration and management of hospitals on the formal side, which can positively affect HBE. These include, among others, brand attitude [6], brand trust [1,4,26,91,94], and customer relationship management (CRM) with knowledge management, long-term association, technology-based CRM, joint problem solving, and customer involvement [99,101].

The effects of marketing tools on HBE were analyzed, indicating, among other things, that distribution and promotion had a direct effect on perceived quality and brand awareness/association and an indirect effect on brand preference. However, there was no significant relationship between distribution-promotion and brand loyalty [3]. Positive effects on HBE were found for E-responsiveness (as the ease and speed of responding online and staying in touch with patients and their families) [98], advertisement [104], customer lifetime value [20], product innovation, process innovation, and service innovation [93], and corporate social responsibility [16,105]. Any new service, management method, method of promotion, or new marketing activity was treated as innovation. Any change that modified the existing operation of the hospital as a whole and its units was treated as an innovation [93]. CSR refers to an organization’s/institution’s commitment to stakeholder interests, sustainability, and improved social conditions. The study considered three dimensions of CSR as ethical, legal, and economic responsibility [16,105].

One study found that tangibles, interaction activity, social responsibility, process expertise, physician’s care, operational activity, service communication, and relationship activity significantly impacted BE [9]. However, it has been shown that ease of use, e-scape, customization [98], safety measures and access convenience [9], and human capital [103] had no significant positive impact on the BE [98].

In turn, BE positive affects the purchase intention towards health services of hospitals [5], including private healthcare organizations [6].

In addition, emergency medical service, especially first-aid activities, educational activities, and medical treatment in emergency rooms, play a significant role in BE for the public health system. On the other hand, rescue/first-aid and transfer activities, educational activities in urgent situations, and medical treatment in emergency rooms influenced brand meaning [95]. In summary, all the determinants of HBE were placed in Table 3, and the studies included in this SLR used various research methods, including SEM, regression analysis, and CFA. This is indicated in Table 1. Therefore, it was impossible to make a uniform quantitative summary. Thus, a map of HBE determinants was prepared, dividing them into traditional HBE determinants, medical-related factors, and other factors (Table 3). The table indicating their direct or indirect impact on brand equity is identified. It is also indicated where the influence of a factor was studied, but no significant statistical relationship was obtained.

It turned out that for hospitals, not only traditional and medical-related factors are studied, but there is also a group of other factors. This has already been shown in Table 2, but here the existence of statistical relationships is indicated.

Among the traditional factors, brand loyalty, perceived quality, brand awareness, brand associations, brand image, and brand familiarity were considered. These factors were analyzed with varying frequency. One of these factors, i.e., brand loyalty, was analyzed in the articles in several approaches, both as attitude loyalty and behavior loyalty. Quality was analyzed both in terms of perceived brand quality and in terms of service quality, which placed it in medical-related factors. Brand familiarity was analyzed in only one article.

The second group of factors was more numerous and diverse. It contained service quality, analyzed as a separate factor, as well as through its components including tangibles, reliability, assurance, empathy, and responsiveness. They showed a direct effect on HBE, and in one case an indirect effect. In this group of factors, there were also factors directly related to the treatment process and provision of medical services, i.e., first-aid activities, disaster response activities, medical treatment in ERs, but also others relating to educational activities and hospital operations, include process organization, infrastructure/physical evidence, atmosphere, or management practices. They have been analyzed less frequently than service quality, which may be due to the different locations of hospitals, different profiles of service provision. In addition, this group of factors has been subject to study more often in recent years.

The largest group turned out to be the third group, and other factors, which can be grouped into other factors related to consumer/patient, brand, marketing, and management. They are discussed in detail in the description to Table 2. In this group, there was the greatest variation because some of these factors were only surveyed once or twice. As with the second group of factors, this group was more often surveyed between 2015 and 2021 than previously.

In addition, some articles analyzed not only the factors that influence HBE, but also considered further relationships, indicating what HBE influences. Therefore, the last part of the table includes those studies that found that HBE influences purchase intentions, customer loyalty, brand loyalty, and brand image.

**Figure 3:**
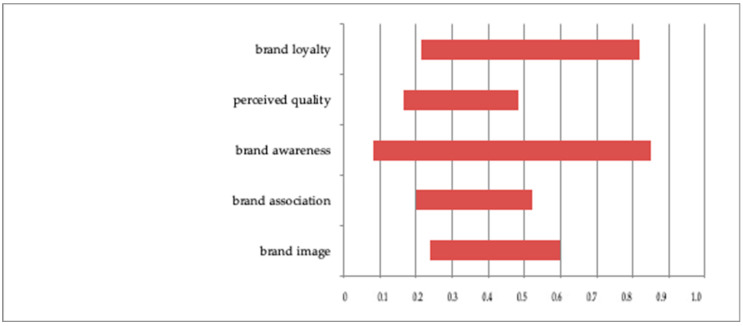
Path coefficient range for traditional determinants in SEM analysis.

As mentioned earlier, it was impossible to make a uniform quantitative summary. For this reason, those studies based on SEM and regression analysis were excluded from Table 3. The results for path coefficients are presented in Figure 3, and the indices for regression analysis are shown in Table 4.

The indices provided indicate the importance of traditional HBE determinants, including brand loyalty, perceived quality, brand associations, brand awareness, and brand image. They indicate specific relationships verified statistically, but it is important to note the variation in their values. The greatest difference in path coefficient was observed for brand awareness, which, regarding hospitals, may be related to patients’ ignorance of the various services provided by hospitals and their ignorance of their rights as patients. The second difference was related to brand loyalty, which, in the case of hospitals, refers to both attitude loyalty and behavioral loyalty.

The results regarding regression analysis show the coexistence of individual factors not only by listing them, but they also show quantitative coefficients. They provide an opportunity to answer the question of which factors can be improved and thus achieve an increase in HBE. However, here we observe variation in both the correlation coefficient and the elements that constitute HBE.

### 4.3. Practical Recommendations

Practical/managerial implications were presented in almost all studies included in the SLR. In Table 5, the practical recommendations are summarized by recommendations for traditional, medical-related, and other factors influencing HBE. In the first group, practical recommendations indicated, for example, the need to improve hospital image and hospital brand [1,2,19,23], maintain or increase brand loyalty [1,3,5,19,23,91,102], and improve perceived quality [3,19,23] and brand awareness [5,103]. In the second group, practical recommendations pointed to the improvement of the hospitals’ facilities and provision of the hospitals’ convenient environment [2], and delivery of qualitative customized services [23].

In the third group, recommendations are related to hospital management and conducting effective marketing activities. One study proposed the introduction of an integrated marketing communication program consisting of two parts. The first part recommended the implementation of training, educational, and public relations programs to increase the level of customer trust, satisfaction, and relationship commitment. The second part focused on launching BE awareness programs for all hospital workers to emphasize the importance of the hospital image for building the hospital image [1].

## 5. Discussion

In this paper, the results of the systematic literature review are presented to analyze factors that determine the HBE. Thirty-two research studies were selected for analysis. Three research questions were formulated.

**In answering the first research question**, it was found that the most frequently analyzed determinant of HBE is loyalty to the hospital brand. This factor was analyzed as brand loyalty [4,5,6,18,19,23,26,90,91,92,96,97,103,106], customer loyalty [106], attitudinal and behavioral loyalty [102], or loyalty [89,92,94]. We have two different understandings of brand loyalty, resulting from the different understandings of brand loyalty in the two classic BE concepts. In Keller’s model, brand loyalty is the outcome of BE [31]. In Aaker’s model, brand loyalty is one of the equivalent elements that comprise and shape BE [30]. The studies included in this SLR considered both approaches. Brand loyalty was treated as a component or determinant of HBE, which was consistent with the theory of D.A. Aaker. There were also those studies that treated brand loyalty as the outcome, in line with K.L. Keller’s theory. However, some studies combined the two concepts. For example, one study examined attitudinal and behavioral approaches and indicated that attitudinal loyalty should be viewed as a source of BE, whereas behavioral loyalty should be perceived as an outcome of BE [102]. In another study conducted in Taiwan, brand loyalty-along with brand awareness, brand association, and service quality-was treated as a component of the brand equity index, indicating that it affects customer loyalty [106].

Brand loyalty is important for hospital services [107,108,109,110,111,112] in various ways. Patients can choose the same hospitals to treat the same diseases, or they can choose the same hospitals in the process of treating other diseases. They can also recommend hospitals to other patients looking for the right place for treatment [113]. With brand loyalty, expanding to more medical disciplines reduces marketing expenses [6]. The greater the loyalty of patients to hospitals, the more the value of the hospital will be appreciated. A loyal patient will be willing to seek service from that hospital again. The hospital will be prioritized over others and can be recommended to other customers, which overall leads to improved brand equity [4,114].

Loyalty to hospitals is determined by, among other things, patient satisfaction [108,109,110,111,112], the quality of services provided [108,109,110,111,112], customer relationship management [110], hospital staff [115], relationship marketing [116], and also issues inherent in the relationship with patients, i.e., patient-physician and patient-hospital communication [115]. This importance of patient loyalty to hospitals fits into the pyramid of brand loyalty. The bottom represents disloyal consumers for whom any brand as suitable. The second level is those who are satisfied with the product or at least not dissatisfied. The next level is satisfied consumers who do not want to risk a product change. The fourth level is loyal consumers who treat the brand as a friend. On the fifth level, some committed consumers are extremely loyal to the brand. They are proud users and will recommend the product to others [30].

In addition, the importance of hospital loyalty is part of a broad understanding of consumer loyalty. Loyal customers are less price sensitive [117], and companies or institutions achieve marketing benefits, i.e., lower financial expenditures on marketing activities [118], positive feedback and recommendations to other customers [119], and increased sales and revenues [120]. This is an element to strengthen the competitive advantage of the company [121].

**In response to the second research question**, it should be noted that the traditional determinants of BE were included in the analysis. They were analyzed in articles published more recently, as well as in earlier ones. Brand loyalty, brand awareness, perceived quality, brand image, and brand associations considered in Aaker’s and K.L. Keller’s concepts were analyzed.

Quality should be discussed separately as it fits into the traditional determinants of HBE (research question 2) as well as refers to specific medical factors describing HBE (research question 3). In a similar way to the concept of D.A. Aaker, perceived quality was analyzed in many of the articles included in our SLR [3,6,23,89,91,103]. In some studies, the concept of perceived quality analyzed patients’ perceptions and assessments of the overall level of quality of services provided, without going into details of medical aspects. Other articles considered several specific variables. For example, a study conducted in India found that the variables that made up the largest component of post-regulated quality were staff attitudes toward patients, staff concern for patients, empathy, communication with patients, and hospital equipment [92]. Dimensions of quality are important, for example, Keller [78] identified seven dimensions of product quality: performance, features, conformation quality, reliability, durability, serviceability, style, and design. In this aspect, the brand should represent a credible guarantee of quality to the consumers [30]. A multi-faceted approach to quality in terms of both perceived quality and service quality demonstrates high importance of quality in the construction of HBE. This is reflected in the literature, where the multidimensional approach to quality also determines how quality is measured as the SERVQUAL Model [122], SERVPERF Service Quality Model [123], Customer Value and Customer Satisfaction Model [124], and INTSERVQUAL Internal Service Quality Model [125].

Brand awareness and brand associations were also analyzed as traditional HBE factors in the articles included in this SLR [3,5,20,26,97,106]. As a rule, they were analyzed together, but their impact on HBE was not always statistically confirmed. In addition, brand awareness analyzed in SEM showed the greatest variation in path coefficient values. An interesting determinant of HBE is brand image [1,2,6,19,23,90,98]. The studies included in this SLR pointed to brand image as a determinant of HBE and gave practical recommendations on how to improve hospital brand image. For example, it was pointed out that improvement of hospital brand image may occur through improving the hospital’s good reputation, improving the hospital’s excellent facilities, provision of the hospital’s convenient environment [2], or positive “word of mouth” [102]. The need for an integrated marketing communication program was also mentioned, including the implementation of training, educational, and public relations programs to increase the level of customer trust, satisfaction, and relationship commitment [1].

**The third research question** concerns the medical-related determinants of HBE. They have been analyzed more frequently in recent years, indicating their increased importance for HBE. They are studied in various ways, both as isolated elements that affect HBE directly and as elements that determine HBE indirectly through their impact on the quality of medical services, associations with the treatment process occurring at a particular hospital, and hospital image, or hospital brand image. This group of factors turned out to be so numerous that a division was made into medical factors directly related to the treatment process and other factors. Among the medical factors ranked were service quality and factors directly related to the treatment process and medical services, i.e., first-aid activities, medical treatment in ERs, but also others relating to educational activities and hospital operations, including, for example infrastructure/physical evidence. Meanwhile, among the other factors, management, marketing, brand, and consumer aspects were studied.

Hospital service quality was analyzed as service quality [100] or patient care service quality [16]. It turned out to be a very important factor and source of HBE due to the fact that it is an evaluation of medical services by patients and indicates patients’ perception of the quality of services provided by the hospital [99,100,103,106]. Hospital service quality was subject to analysis as a single factor expressing the patient’s or consumer’s judgment about a service’s overall excellence or superiority [75] or as a collection of components, including tangibles, reliability, responsiveness, assurance, and empathy [100]. In one article from this SLR, it was proven that the way of examining the quality of medical services should consider specific elements and it is not necessary to adapt commonly known methods of examining service quality. Therefore, the 5Qs model of service quality was adapted to assess the quality of medical services. It includes five elements, i.e., (1) object quality, which is the technical quality of services relating to clinical procedures; (2) treatment process quality, related to functional quality, which describes how health services are delivered; (3) infrastructure quality, which are the skills, competence of staff, and assurance of prompt delivery of health services; (4) interaction quality in terms of information exchange, financial exchange, and social exchange; and (5) atmosphere quality, related to the friendliness or relationship with the service provider [90].

Hospital service quality, that makes patients know, remember, revisit, and recommend a hospital to others includes not clean rooms and departments, advanced medical equipment, and effective procedures, but also knowledgeable and caring clinicians who provide patients with physical and emotional relief and disease knowledge during the hospital treatment process and in post-treatment life. This inspires patients and their families to associate their treatment needs with a particular hospital brand resulting in high brand loyalty [16]. The healthcare service quality aspects (i.e., physical environment, customer-friendly environment, responsiveness, communication, privacy, and safety) are positively related to patient loyalty which is mediated through patient satisfaction [111].

In recent years, empirical studies on HBE have reported on consumer/patient, brand (other than traditional), marketing, and management factors other than those described above. These have been classified as other determinants of HBE. A wide set of these factors have been analyzed, including patient satisfaction [2], emergency medical service [95], health care utilization [5], physical aspect, staff attributes [92], or examining the quality of health care services [16]. For example, in a study conducted in South Korea, first-aid activities, disaster response activities, educational activities, and medical treatment in emergency rooms were considered as emergency medical services [95]. In contrast, a study conducted in India considered physical environment (atmosphere, tangibles, infrastructure facility), interpersonal care activity (interaction activity, relationship activity, physician’s activity), technical process (process expertise, safety measures), and administrative procedure (timeless of activity, operational activity) [9]. In another study, patient experience was considered as a sensory, affective, behavioral, and intellectual experience [9]. Patient satisfaction was analyzed from the point of view of meeting patients’ needs in terms of satisfaction with nursing service, satisfaction with the use of medical instruments, satisfaction with administrative service, and willingness to use hospital services again [2].

In recent years, issues related to corporate social responsibility [103] and social responsibility [9] have also been incorporated into HBE research. Individual dimensions of CSR (ethical, legal, and economic) have also been examined [15,105]. For example, a study of patients and middle management employees in Vietnam examined the impact of three CSR domains (ethical CSR, legal CSR, and economic CSR) and two leadership styles (transformational and transactional) on HBE. It was pointed out that brand equity is analyzed from the perspective of patients whose needs in the treatment process, the way treatment is organized, and the quality of services provided, depend on hospital management. Therefore, it concluded, hospital directors should consider their leadership style to achieve synergy with CSR [105]. A consumer perspective was taken into account, pointing to the customer experience (sensory, affective, behavioral, intellectual) [9], customer lifetime value [20], and customer relationship management [99,103], with a focus on components, i.e., IT infrastructure, human capital, organizational architectural framework, quality of service [103].

Some studies have examined the influence of marketing factors on HBE. All elements of the marketing mix for services have been considered i.e., price, distribution, promotion, physical evidence, people, process [103], and integrated marketing communication (advertising, continual medical education programs, public relations, online media, word of mouth) [17] or only some elements, including word of mouth [98] or advertising [104]. However, no clear correlations were obtained for the impact of marketing activities on HBE. This may be because the direct impact of price and distribution was analyzed, whereas others had a broader spectrum that fits into an integrated marketing communication process. Where price was part of the marketing communication or loyalty program, an impact on HBE was obtained.

The differentiation of HBE determinants has been observed in recent years in the context of public and private hospitals [20,95]. A study conducted in South Korea analyzed emergency medical service via a patient-centered approach in four areas: rescue/first-aid and transfer activities, disaster prevention, preparation, and response activities, educational activities in urgent situations, and medical treatment in emergency rooms. It said that the public health system must be considered as a part of the governance structure emergency medical service, especially first-aid activities, educational activities, and medical treatment in ERs, which all play a significant role in brand equity for the public health system [95]. In contrast, a study of public and private hospital patients in Indonesia found that brand equity was the dominant variable for increasing customer lifetime value in the public case, whereas private hospitals showed no significant difference. These are single articles that consider the division between public and private hospitals, but the different characteristics of public and private hospitals led to different market responses [20]. In addition, as stated in the introduction, brand equity is essential in government sectors, as it can increase the public’s credibility, trust, and loyalty to the government [27] as well as empathy and understanding of patients’ needs [28].

This is a direction for future research in identifying the HBE determinants of public and private hospitals, but also in understanding patient needs and perceptions of service quality and the overall treatment process.

## 6. Conclusions

Based on this SLR, it is important to emphasize that HBE is determined by various factors, the number of which has been increasing recently. In addition, there has been more research on HBE in recent times. This is because HBE is treated as a value perceived by the patient in the context of his or her own health. There are traditional determinants of HBE (perceived quality, brand image, brand awareness, and brand associations), medical factors related to patients’ perceptions of the quality of services provided, and those relating to the operation of hospitals and the implementation of the treatment process. There are also other factors relating to patient satisfaction, patient experience, social responsibility, management processes, undertaking effective marketing communications, and creating relationships with patients. This shows that in recent years, with the changes in the environment and the increase in patient awareness, it is not only the treatment process, physicians’ knowledge, and specialized equipment that are important for HBE, but also the approach to patients, the creation of relationships with them, empathy as a component of service quality, etc. These factors take on additional significance if we analyze not only the hospital treatment process itself, but various aspects of public health, including prevention, improving quality of life, health policy, and health care law and governance.

This SLR fills a gap in terms of publications on HBE. It indicates a recent increase in the diversity of HBE determinants and points to practical recommendations both in terms of brand equity, service quality, and healthcare delivery processes, and also in terms of better understanding of patients’ needs and their perceptions of healthcare services. However, it has limitations on the exclusion criteria used for not considering conference materials, books, dissertations, and others. However, this is due to the rules applicable to all SLRs.

This type of research on HBE should be continued by trying to identify HBE determinants and introduce quantitative indicators to compare BE of different types of hospitals, private and public, and changes over time should be analyzed. This may proven to be particularly important for understanding the needs and desires of patients and perceptions of service quality and the overall treatment process. Research should be carried out in the form of a systematic literature review and empirical studies among patients of public and private hospitals. Furthermore, the issue of the quality of medical services should be studied. This will improve the quality of medical services and promote preventive healthcare, which will have an impact on public health.

## Figures and Tables

**Table 1:** General details of studies included in the systematic survey.

Author, Year	Research Method	City, Country	Sample Population	Methods
Sukawati (2021) [2]	Survey	Denpasar, Bali, Indonesia	81 patients	Regression analysis
Fong, et al. (2021) [6]	Survey	Malaysia	271 patients	PLS-SEM
Kim, et al. (2021) [95]	Survey	South Korea	150 patients	CB-SEM
Ozkoc, et al. (2020) [3]	Survey	Iran, Istanbul, Ankara	500 patients	CB-SEM
Kalhor, et al. (2020) [4]	Survey	Iran	450 patients	CB-SEM
Ernawaty, et al. (2020) [5]	Survey	Airlanga, Indonesia	381 patients	Multiple logistic regression
Adhyka, et al. (2019) [97]	Survey	Indonesia	115 patients	PLS-SEM
AlSaleh (2019) [98]	Survey	Kuwait	232 patients	CB-SEM
Shriedeh et al. (2019) [99]	Survey	Jordan	454 patients	CB-SEM
Sudirman, et al. (2018) [20]	Interviews	Makassar, Indonesia	60 respondents	Regression analysis
Roy et al. (2018) [89]	Face-to-face interview	India	90 patients (Bangladesh, Bhutan, China, Taiwan)	Content analysisCluster analysis
Mukaram et al. (2018) [18]	Survey	Indonesia	905 patients	Regression analysis
Kumar et al. (2018) [9]	Survey	India	839 patients	CB-SEM
Altaf, et al. (2018) [90]	Survey	Pakistan	393 patients	PLS-SEM
Srikanth, et al. (2017) [17]	Survey	India	300 patients	CB-SEM
Shriedeh, et al. (2017) [100]	Survey	Amman, Jordan	306 patients	CB-SEM
Shriedeh, et al. (2017) [101]	Survey	Amman, Jordan	306 patients	CB-SEM
Feiz, et al., (2016) [90]	Survey	Teheran, Iran	388 patients	CB-SEM
Tiwari, et al. (2016) [91]	Survey	India	150 patients	Factor analysis
Shriedeh (2016) [92]	Survey	Amman, Jordan	339 patients	CB-SEM
Azarnoush, et al. (2016) [94]	Survey	Iran	380 patients	CB-SEM
Piaralal, et al. (2015) [19]	Survey, self-administered questionnaires	Klang Valley, Malaysia	123 patients	Regression analysis
Lingavel (2015) [103]	Survey	Sri Lanka	127 patients	Regression analysis
Charanah, et al. (2015) [104]	Survey	Nairobi, Kenya	74 employees (administration and marketing)	Regression analysis
Tuan (2014) [16]	survey self-administered questionnaires	Vietnam	417 patients	PLS-SEM
Kumar, et al. (2013) [26]	qualitative in-depth interviews	India	902 patients	CB-SEM
Karbalaei, et al. (2013) [96]	Survey	Teheran, Iran	318 patients	PLS-SEM
Chahal, et al. (2012) [23]	Survey	Jammu city, India	206 patients	Regression analysis CB-SEM
Tuan (2012) [105]	survey, self-administered structured questionnaires	Vietnam	226 middle-level managers and 714 patients	CB-SEM
Wang, et al. (2011) [106]	Survey	Taiwan	250 patients	PLS-SEM
Chahal, et al. (2010) [102]	Survey	Jammu, India	300 patients	EFA, CFA
Kim, et al. (2008) [1]	Survey	South Korea	532 patients	CB-SEM

CB-SEM—covariance based structural equation modeling, PLS-SEM—partial least squares based structural equation modeling.

**Table 2:** Determinants of HBE and key findings.

Author, Year	Traditional Determinants of HBE	Medical-Related Determinants of HBE	Other Determinants of HBE	Key Findings
Sukawati (2021) [2]	brand image	hospital service quality, patient satisfaction		Hospital brand image positively and significantly influences patient satisfaction and service quality. Service quality positively and significantly influences patient satisfaction.
Fong, et al. (2021) [6]	perceived quality brand loyalty brand awareness brand associations brand image		brand attitude	Perceived quality, brand loyalty, and brand attitude positively and significantly influence BE. Brand image, brand awareness, and brand associations show no relationship with BE. BE positive influences the purchase intention of health services provided by private healthcare organizations.
Kim, et al., (2021) [95]		first-aid activities, disaster response activities, educational activities, medical treatment in emergency rooms, governance perception	brand meaning brand response brand relationship	The perceived level of governance between local and central government influences the relationship between functions of emergency medical service, and brand meaning of the public health service. Emergency medical services, especially first-aid activities, educational activities, and medical treatment in emergency rooms, play an important role in BE for the public health system. The activities related to rescue/first-aid, educational activities, and medical treatment in ERs are presented more frequently and are in closer proximity than disaster prevention, preparation, and response activities. Rescue/first aid and transfer activities, educational activities in urgent situations, and medical treatment in emergency rooms influenced brand meaning. Brand meaning influenced brand response and brand response influenced brand relationship.
Ozkoc, et al. (2020) [3]	brand loyalty perceived quality brand awareness brand associations	physical evidence, people (hospital staff), process of providing medical services	brand preference price distribution promotion brand management practices	Price has a direct negative effect on brand loyalty and brand preference. There is no relationship between price-perceived quality and price-brand awareness/association. Distribution in hospitals is related to access to health services. Distribution-promotion has a direct effect on perceived quality and brand awareness/association and indirect effect on brand preference. However, there is no significant relationship between distribution-promotion and brand loyalty. Price and promotion have no effect on BE dimensions, neither direct nor an indirect effect on brand preference. Physical evidence has a direct effect on BE dimensions and has an only indirect effect on brand preference. People have a direct effect on BE dimensions. Properly functioning processes shape patient perceptions of quality and influence hospital preferences.
Kalhor, et al. (2020) [4]	brand loyalty perceived quality brand associations brand awareness		brand trust brand satisfaction	There is a relationship between brand trust and brand loyalty and BE.
Ernawaty, et al. (2020) [5]	brand loyalty brand awareness brand associations	patient visits		BE influences patient visits. Brand awareness, brand associations, and brand loyalty influence patient visits. The brand associations have the greatest impact among the three attributes.
Adhyka, et al. (2019) [97]	brand loyalty brand awareness brand associations perceived quality		word of mouth purchase intention	Brand awareness, brand association, perceived quality, and brand loyalty are important dimensions in building BE. BE and word of mouth have a significant impact on patient purchase intentions in hospitals.
AlSaleh (2019) [98]	brand loyalty brand awareness brand image	customization security	e-responsiveness ease of use e-scape	CRM and service quality are significant predictors of overall BE and have further strengthened the role of service quality as a key mediating variable. CRM is an important factor influencing service quality. Service quality is a source of overall BE.
Shriedeh et al. (2019) [100]		service quality: tangibility, reliability, responsiveness, assurance, empathy	CRM: knowledge management, customer involvement, long-term association, technology-based CRM, joint problem solving	E-responsiveness and security have a strong and direct influence on BE Ease of use, e-scape, and customization have no significant positive impact on the BE.
Sudirman, et al. (2018) [20]	brand loyalty perceived quality brand awareness brand association		value equity, retention equity customer lifetime value	BE is the dominant variable to increase the customer lifetime value for public hospitals, whereas there is no significant difference for private hospitals.
Roy et al. (2018) [89]	brand awareness, perceived quality brand quality brand loyalty	infrastructure (hospital)	infrastructure (country) culture	Brand awareness, brand association/destination association, destination perceived quality, loyalty, culture, and destination infrastructure contribute positively to consumer-based BE for medical tourism.
Mukaram et al. (2018) [18]	brand awareness brand association quality perception brand loyalty		buying decision	Brand awareness, brand association, and brand loyalty as variables of BE influence the purchase decision of hospital services. Quality perception does not affect the purchasing decision of hospital services.
Kumar et al. (2018) [9]		Physical environment (atmosphere, tangibles, infrastructure facility) Interpersonal care activity (interaction activity, relationship activity, physician’s care) Technical process (process expertise, safety measures) Core service, service charge, Customer experience (sensory, affective, behavioral, intellectual)	Administrative procedure (timeless of activity, operational activity) social responsibility, service communication, access convenience	Tangibles, interaction activity, social responsibility, process expertise, physician’s care, operational activity, service communication, and relationship activity significantly positively influence customer experience. Safety measures and access convenience prove to have a significant negative impact on customer experience. Atmosphere, infrastructure facility, timeliness of activity, core service, and service charges have no significant effect on customer experience.
Altaf, et al. (2018) [90]	brand awareness brand image brand loyalty	5Qs model of health-care service quality (HCSQ): quality of object, treatment process, infrastructure, interaction, atmosphere		Health care service quality has a weak relationship with hospital brand loyalty, but a strong relationship with brand image and brand awareness. Brand awareness and brand image have a strong relationship with brand loyalty. Brand image and brand loyalty have a strong relationship with overall HBE, but a non-significant relationship was found between brand awareness with overall HBE.
Srikanth, et al. (2017) [17]	brand awareness brand image		Integrated marketing communication (advertising, continual medical education programs, public relations, online media, word of mouth, SMS	Integrated marketing communication affects brand awareness and brand image, in turn, brand awareness determines HBE. No effect of brand image on HBE was found.
Shriedeh, et al. (2017) [100]		service quality: tangibles, reliability, responsiveness, assurance, and empathy		Each of the service quality dimensions that relate to tangibility, reliability, responsiveness, empathy, and assurance are significantly correlated with overall BE.
Shriedeh, et al. (2017) [101]			CRM: knowledge management, long-term association, technology-based CRM, joint problem solving, customer involvement,	The customer relationship dimensions (customer involvement, long-term association, and joint problem solving) have a significant and positive impact on overall BE. Knowledge management and technology-based customer relationship management have insignificant effects on overall BE.
Feiz, et al., (2016) [91]	brand associations perceived quality brand trust brand loyalty brand awareness		relationship commitment	HBE was influenced by brand associations, perceived quality, brand trust, relationship commitment and brand loyalty. A positive effect of brand awareness on brand associations, brand associations on perceived quality, perceived quality on brand trust, brand trust on relationship commitment, and relationship commitment on brand loyalty was observed. The effect of brand awareness on BE was insignificant.
Tiwari, et al. (2016) [92]	perceived quality brand loyalty brand image	staff attribute, physical aspects		Six subdimensions contribute to the three major components of BE, of which perceived quality has the greatest impact on BE. The physical aspect, which includes lighting, drinking, transportation, physical, security, sewerage, medical record, medical facility, and staff attribute belongs to the perceived quality component of the HBE. The loyalty aspect, which includes service trust, a positive and clean environment, and switching aspect, belongs to the brand loyalty component of the HBE. In turn, brand value and value for money determine the brand image.
Shriedeh (2016) [93]			Innovations: product, process, service, administrative, marketing	Innovation contributes significantly to BE. Product, process, and service innovations positively and significantly affect overall BE. Administrative and marketing innovations do not have a significant impact on overall BE.
Azarnoush, et al. (2016) [94]	brand loyalty	service quality	satisfaction, brand trust commitment, tendency to maintain the relations, experience	HBE is directly influenced by patient satisfaction, experience, and loyalty to the hospital brand. Trust, willingness to maintain the relations and commitment are the other factors that positively affect patient loyalty. The factor of ‘previous experiences’ has no significant impact on hospital loyalty.
Piaralal, et al. (2015) [19]	perceived quality brand loyalty brand image			There is a strong relationship between BE and perceived quality, brand loyalty, and brand image.
Lingavel (2015) [103]	brand associations brand awareness, perceived quality brand satisfaction brand loyalty		CRM: information technology infrastructure, human capital, organizational architectural framework, quality of service	Customer relationship management has an impact on BE. Information technology, organizational architecture, and service quality in customer relationship management significantly contribute to BE. There is a negative relationship between human capital and BE.
Charanah, et al. (2015) [104]			Advertisement parameters: frequency, budget	Advertising influences HBE. The frequency of advertisement activities increases brand awareness leading to greater HBE
Tuan (2014) [16]	Brand loyalty perceived quality brand awareness brand associations	Clinical governance effectiveness Patient care service quality (reliability, assurance, empathy, responsiveness, tangibles)	CSR dimensions (ethical CSR, legal CSR, economic CSR)	Ethical CSR was found to have a positive relationship with clinical governance effectiveness. Legal CSR or economic CSR does not promote clinical governance effectiveness as evidenced by the negative and significant relationships between legal CSR and clinical governance effectiveness, and between economic CSR and clinical governance effectiveness. Clinical governance would be positively associated with reliability, assurance, empathy, responsiveness, or tangibles. Positive and significant relationships were observed between service quality dimensions (reliability, assurance, empathy, responsiveness, or tangibles and BE).
Kumar, et al. (2013) [26]	brand awareness brand association perceived quality brand loyalty		brand trust, brand experience dimensions: sensory, affective, behavioral, and intellectual	The brand experience dimensions positively influence the five BE dimensions. BE dimensions (brand awareness, brand association, perceived quality, brand trust, and brand loyalty) influence customer-based HBE.
Karbalaei, et al. (2013) [96]	brand loyalty brand awareness	customer satisfaction hospital image	relationship commitment trust,	Trust, customer satisfaction, and relationship commitment have a positive impact on brand loyalty and brand awareness. Brand awareness and brand loyalty significantly positively influence BE. BE had a significant positive influence on hospital image. Trust, customer satisfaction and relationship commitment also have a significant positive influence on hospital image.
Chahal, et al. (2012) [23]	brand loyalty brand image	Service BE in the healthcare sector: service quality, staff, behaviour, tangibles)		BE of healthcare services is highly influenced by brand loyalty and perceived quality. Brand image has an indirect effect on service BE through brand loyalty
Tuan (2012) [105]	brand loyalty brand association brand awareness perceived quality		CSR dimension: ethical, legal, economic Leadership styles transformational, transactional	Transformational and transactional leadership is significantly related to ethical CSR, legal CSR, economic CSR, and BE. Ethical CSR influences BE, but economic and legal CSR do not influence BE.
Wang, et al. (2011) [106]	brand awareness brand associations brand loyalty	service quality	customer loyalty	BE is determined by brand awareness, brand associations, service quality, and brand loyalty. The level of BE influences customer loyalty.
Chahal, et al. (2010) [102]	attitudinal loyalty as a source of BE		behavioral loyalty as an outcome of BE	BE is directly influenced by consumer attitude, which is reflected in their behavior. Attitudinal loyalty and behavioral loyalty were accepted as the indicators of BE. BE reflects attitudinal loyalty and behavioral loyalty. The four attitudinal loyalty indicators support the idea that patients choose a hospital based on an important criterion such as staff expertise, availability of state of art technology, hospital performance and overall hospital performance.
Kim, et al. (2008) [1]	brand loyalty brand awareness	hospital image	trust customer satisfaction relationship commitment,	Trust, customer satisfaction, and commitment to the customer relationship have a positive impact on brand loyalty and brand awareness. Brand awareness has a significant positive impact on BE, whereas brand loyalty has no such impact. BE had a significant positive influence on hospital image. Trust, customer satisfaction, and relationship commitment also have significant positive effect on hospital image.

**Table 3:** Map of all HBE determinants.

Author, Year	Traditional Determinants of HBE						Medical-related Determinants of HBE																Other Determinants of HBE																								Influence of HBE			
	Brand Loyalty	Perceived Quality	Brand Awareness	Brand Associations	Brand Image	Brand Familiarity/Attitude	Service Quality:	Tangibles	Reliability	Assurance	Empathy	Responsiveness	First-Aid Activities	Disaster Response Activities	Educational Activities	Medical Treatment in ERs	Process	Atmosphere	Infrastructure/Physical evidence	Activities/Management/Practices	Physicians care/Staff	Clinical Governance Effectiveness	Brand/Customer Satisfaction	Brand Trust	Brand/Customer Experience	Brand Preference	Brand Identity	Brand Meaning	Brand Response	Brand Relationship	Relationship Commitment	Corporate Social Responsibility	Leadership styles	Advertisement	Word of Month	Integrated Marketing Communication	Customer Relationship Management:	Security	Customization	Ease of Use and e-Scape	E-Responsiveness	Culture	Customer Lifetime Value	Elements of Marketing Mix	Administrative and Marketing Innovations	Service Innovations	Customer Loyalty	Brand Loyalty	Hospital Image	Purchase Intention/Decision
Sukawati (2021) [2]																																																		
Fong, et al. (2021) [6]																																																		
Kim, et al., (2021) [95]																																																		
Ozkoc, et al. (2020) [3]																																																		
Kalhor, et al. (2020) [4]																																																		
Ernawaty, et al. (2020) [5]																																																		
Adhyka, et al. (2019) [97]																																																		
AlSaleh (2019) [98]																																																		
Shriedeh et al. (2019) [100]																																																		
Sudirman, et al. (2018) [20]																																																		
Roy et al. (2018) [89]																																																		
Mukaram et al. (2018) [18]																																																		
Kumar et al. (2018) [9]																																																		
Altaf, et al. (2018) [90]																																																		
Srikanth, et al. (2017) [17]																																																		
Shriedeh, et al. (2017) [100]																																																		
Shriedeh, et al. (2017) [101]																																																		
Feiz, et al., (2016) [91]																																																		
Tiwari, et al. (2016) [92]																																																		
Shriedeh (2016) [93]																																																		
Azarnoush, et al. (2016) [94]																																																		
Piaralal, et al. (2015) [19]																																																		
Lingavel (2015) [103]																																																		
Charanah, et al. (2015) [104]																																																		
Tuan (2014) [16]																																																		
Kumar, et al. (2013) [26]																																																		
Karbalaei, et al. (2013) [96]																																																		
Chahal, et al. (2012) [23]																																																		
Tuan (2012)[105]																																																		
Wang, et al. (2011) [106]																																																		
Chahal, et al. (2010) [102]																																																		
Kim, et al. (2008) [1]																																																		

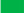
 Factor having a direct effect on HBE (statistically significant). 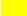 Factor having an indirect effect on HBE (statistically significant). 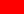 Factor studied but not influenced either directly or indirectly by HBE. 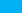 HBE as a factor affecting other factors.

**Table 4:** Regression analysis for traditional HBE determinants.

Study	R^2^	Regression
Mukaram, Sangen, Rifani, 2018	0.96	HBE = −5.295 + 0.691 × BL + 0.147 × PQ + 0.067 × Baw + 0.081 × BAss
Piaralal, Mei, 2015	0.52	HBE = −0.317 + 0.2.65 × BL + 0.465 × PQ + 0.333 × BI
Lingavel, 2015	0.68	HBE = 0.512 + 0.154 × Human Capital + 0.284 × IT + 0.146 × SQ + 0.269 × OA
Charanah, Njuguna, 2015	0.33	n.a
Chachal, Bala, 2012	0.38	HBE = n.a. + 0.393 × BL + 0.31 × PQ − 0.036 × BI

BL—brand loyalty, PQ—perceived quality, Baw—brand awareness, BAss—brand associations, BI—brand image, OA—organizational architecture, R^2^—R-squared as a goodness-of-fit measure for regression models.

**Table 5:** Practical implications by groups of HBE determinants for the studies included in the systematic survey.

Author, Year	Traditional HBE Determinants	Medical-Related HBE Determinants	Other HBE Determinants
Sukawati (2021) [2]	To improve the brand image of the hospital by improving its good reputation, its facilities, and providing a convenient environment. To improve brand image by providing good services, so that patient satisfaction is maintained.		
Kim, et al. [95]	To build strong loyalty to the public health system by improvements in first aid, education, and medical treatment in emergency rooms To create a favorable brand image and public loyalty to the public health system by managing an effective management structure between the central and local governments	To increase satisfaction with various functions of emergency medical service. To ensure systematic cooperation between the central government and local governments by supporting educational activities in emergency rooms or monitoring the needs of local governments.	To manage an effective management structure between the central and local governments to create a favorable brand image and public loyalty to the public health system.
Ozkoc, et al. (2020) [3]	To improve brand loyalty and brand preference by using a price strategy, To increase perceived quality and preference by improving hospital processes.	To motivate hospital employees To improve physical evidence (hospital lighting, ventilation, cleaning, equipment in working conditions, employee clothing, etc.)	
Kalhor, et al. (2020) [4]	To give priority to the dimensions and drivers of BE to maintain their place in society and provide effective services.		
Ernawaty, et al. (2020) [5]	To increase BE and healthcare utilization by promotion to create familiarity and, good impression To build brand awareness, provide good services to increase brand association, and maintain brand loyalty by enhancing interactions with patients,	To ensure constant direct contact with patients and periodically measure patient satisfaction.	
Adhyka et al. (2019) [98]	To build strong BE dimensions in the highly competitive hospital services market.		
Shriedeh et al. (2018) [100]			To adjust strategic factors to build strong medical tourism brands with greater emphasis on delivering higher levels of service quality.
AlSaleh (2019) [98]		To ensure by hospital managers and employees that the process of providing services involves a high level of security and trust. To offer high-level training to hospital staff that emphasize the importance of safety and trust.	To develop training centers for the hospital managers and organize education and training sessions that focus on responding quickly to customer needs.
Sudirman, et al. (2018) [20]	To strengthen BE created in a public hospital to increase market share, reduce promotion costs, and increase customer equity		To improve competitive excellence in an era of growth in the healthcare industry
Roy et al. (2018) [89]			To provide a standard guideline for hospital tourism managers.
Mukaram et al. (2018) [18]			To engage by hospital management in community activities such as corporate social responsibility To be aware of competitors’ innovations, especially in product development due to increasing competition in hospital services
Kumar et al. (2018) [9]		To strengthen managers’ awareness that properly designed tangibles, interaction activities, social responsibility, process knowledge, physician care, operation activities, service communications, and relationship activities of the hospital evoke positive experiences in customers through personal transformation.	To strengthen managers’ awareness that in addition to treating disease, they are also selling an experience that are triggered by the company’s activities. To improve billing, discharge, and other administrative activities, To improve communication of facilities and service successes to customers.
Altaf, et al. (2018) [90]		To analyze the quality of healthcare and emergency services in private cardiology hospitals.	
Srikanth, et al. (2017) [17]	To increase perceived brand awareness and brand image by creating strong integrated marketing communication.		To focus marketing efforts on effective brand management. To implement integrated marketing communication strategies (advertising, public relations, patient communication) through continuing medical education programs, service training, and online marketing.
Shriedeh, et al. (2017) [100]	To build strong brands that are viewed favorably by customers.	To provide high-quality services to customers.	To create a unique customer experience environment.
Shriedeh, et al. (2017) [101]			To improve CMR as one of the most competitive strategies to strengthen BE and increase the competitive advantage of medical tourism.
Feiz, et al., (2016) [91]	To pay attention to the factors influencing HBE To take the necessary measures to increase hospital loyalty and HBE management.		
Shriedeh (2016) [93]		To invest in technological health products, ease of operational processes, and service activities toward positive perceptions.	
Azarnoush, et al. (2016) [94]	To increase patients’ loyalty and trust in the quality of services.	To improve relationships with patients during hospitalization and after hospital discharge by appropriate strategies included in hospital policies.	
Piaralal, et al. (2015) [19]	To monitor the determinants of BE: perceived quality, brand loyalty, and brand image and keep up with the needs of patients.	To manage patient perceptions of hospital services, quality, and outcomes.	
Langavel (2015) [103]	To increase customer awareness of the medical services provided by the hospital.	To identify and stimulate employee talents and skills. To develop various technical, business management, and entrepreneurial skills of hospital staff. To acquire the skills and knowledge of the hospital staff on action plans.	To increase awareness and understanding of management processes.
Charanah, et al. (2015) [104]			To develop realistic advertising to inform the public about the hospital services.
Tuan (2014) [16]		To guide clinicians by clinical leaders (the chief executive officer—CEO, chief medical officer—CMO, and medical officer) to be accountable to all other stakeholders with an emphasis on sustainable community health. To provide training and coaching for nurses to raise awareness so that clinical care is not “too impersonal” for patients (as part of the clinical governance mechanism).	To change the behavioral patterns of clinical faculty members, elevating their responsibility beyond economic and legal CSR to ethical CSR, in which clinicians not only treat patients’ illnesses but also guide them to be physicians or nurses. To be open to direct feedback from patients and nurses.
Karbalaei, et al. (2013) [96]	To build a positive image through proper BE management	To take care of patients well enough that patients develop trust in the hospital, feel satisfied with it, and create a high level of commitment to the hospital.	
Chahal, et al. (2012) [23]	To create, enhance, and maintain service BE through service quality To create brand loyalty to sustain competitive advantage.	To focus on staff behavior, assurance, and tangibility.	
Tuan (2012) [105]			To implement ethical CSR initiatives (charity check-up, charity surgery, and health programs), for a competitive position in the marketplace and a successful and differentiated BE
Wang, et al. (2011) [106]	To understand and measure the BE		
Chahal, et al. (2010) [102]	To build a good image of a hospital by positive “word of mouth” To strengthen attitudinal loyalty with some unique associations (expertise skill of the staff, availability of state-of-the-art equipment, functioning, and overall performance of the hospital), which creates and builds positive perceptions and ultimately influences its behavior. To increase loyalty in terms of attitude and behavior by good expertise skill of the staff, technical facilities available, image of the hospital in providing quality customized services as these factors		To build trust and positive feelings towards the hospital.
Kim, et al. (2008) [1]	To learn how to link brand loyalty with BE. To create a strong HBE by implementing training, educational, and PR programs to increase customer trust, satisfaction, and relationship commitment To create a positive hospital image by launching BE awareness programs for hospital employees, educating them on the important relationship between BE and hospital image.		To create and maintain strong customer relationships to increase customer commitment. To focus marketing efforts on customers with a high level of trust in hospital service hoping that this will lead to a positive BE and hospital image.

## Data Availability

Data are available at the Dapartmnt of Food Market and Consumption Research in the Institute of Human Nutrition Sciences, Warsaw University of Life Sciences, in Poland.
